# The malate–aspartate shuttle (Borst cycle): How it started and developed into a major metabolic pathway

**DOI:** 10.1002/iub.2367

**Published:** 2020-09-11

**Authors:** Piet Borst

**Affiliations:** ^1^ Division of Cell Biology The Netherlands Cancer Institute Amsterdam The Netherlands

**Keywords:** aspartate, citrate‐malate cycle, glycerol‐1‐P cycle, inborn errors, MAS, NADH/NAD ratio, reductive carboxylation

## Abstract

This article presents a personal and critical review of the history of the malate–aspartate shuttle (MAS), starting in 1962 and ending in 2020. The MAS was initially proposed as a route for the oxidation of cytosolic NADH by the mitochondria in Ehrlich ascites cell tumor lacking other routes, and to explain the need for a mitochondrial aspartate aminotransferase (glutamate oxaloacetate transaminase 2 [GOT2]). The MAS was soon adopted in the field as a major pathway for NADH oxidation in mammalian tissues, such as liver and heart, even though the energetics of the MAS remained a mystery. Only in the 1970s, LaNoue and coworkers discovered that the efflux of aspartate from mitochondria, an essential step in the MAS, is dependent on the proton‐motive force generated by the respiratory chain: for every aspartate effluxed, mitochondria take up one glutamate and one proton. This makes the MAS in practice uni‐directional toward oxidation of cytosolic NADH, and explains why the free NADH/NAD ratio is much higher in the mitochondria than in the cytosol. The MAS is still a very active field of research. Most recently, the focus has been on the role of the MAS in tumors, on cells with defects in mitochondria and on inborn errors in the MAS. The year 2019 saw the discovery of two new inborn errors in the MAS, deficiencies in malate dehydrogenase 1 and in aspartate transaminase 2 (GOT2). This illustrates the vitality of ongoing MAS research.

**Abbreviations**
GAPDH: glyceraldehyde‐3‐P dehydrogenase
GOT1/2: glutamate oxaloacetate transaminase 1/2 (aspartate transaminase)
IDH 1,2,3: isocitrate dehydrogenase 1,2,3
LDH: lactate dehydrogenase
MAS: malate–aspartate shuttle
MDH 1,2: malate dehydrogenase 1,2
NAD(P): nicotinamide adenine dinucleotide (phosphate)
PARP: poly‐ADPribose polymerase
PI3K: phosphatidylinositol 3‐kinase

## 1 INTRODUCTION

As a student, I studied the properties of tumor mitochondria in Ehrlich ascites tumor cells. In vain, I tried to find anything abnormal. They were certainly not uncoupled, as postulated by the formidable German Nobel laureate Otto Warburg. In looking for more fruitful topics, I ended up with the malate–aspartate shuttle (MAS).^1^ At the time I was a student in the lab of Bill Slater,^2^ one of the giants of the early history of mitochondrial metabolism and oxidative phosphorylation (OXPHOS), Slater had formulated the chemical hypothesis for the mechanism of OXPHOS (see Reference 2), which was generally accepted before Peter Mitchell entered the scene with his revolutionary chemi‐osmotic hypothesis. For a further refinement of his hypothesis, Slater often asked a student to check a detail, in my case whether the respiratory chain required also inorganic phosphate (Pi) when it was uncoupled from ATP synthesis by the addition of the uncoupler dinitrophenol. This was an important detail, because it was not known at the time whether the postulated high‐energy intermediate in OXPHOS contained phosphate. I checked this with rat‐liver mitochondria oxidizing glutamate—the favorite substrate of the time—with clear‐cut results: glutamate oxidation by isolated mitochondria required Pi, even when the mitochondria were completely uncoupled. This result was sent off to Nature and published without delay.^3^ This may seem incredible today, but the time was 1959. High‐impact journals had not been invented yet and substantial experimental work was sent to BBA, JBC, or Biochem. J. Only tidbits were sent to Nature and published without peer‐review, because the editor did not consider that necessary for papers from reputable labs.^4^

Soon after the publication of this Nature letter, the Slater lab was visited by Feodor Lynen, Nobel laureate, and shrewd biochemist. Although famous for his work on fatty acid and cholesterol biosynthesis, he had also done other work on mitochondrial metabolism and he was a real mito‐insider. Slater had the excellent habit of inducing his prominent visitors to sit down with one of his students. I was the lucky one and Lynen complied. He looked in detail at my experiments on the Pi requirement of glutamate oxidation and then pointed out that there was an unproven assumption in my experiments: at the time, everybody in the mitochondrial field assumed that glutamate was mainly oxidized to 2‐oxoglutarate via glutamate dehydrogenase and that the further oxidation of 2‐oxoglutarate was negligible. If this assumption were wrong, however, this would explain the need for Pi in glutamate oxidation, as the substrate‐linked phosphorylation step following 2‐oxoglutarate oxidation, the succinyl‐CoA synthetase reaction, requires Pi. This was one of the biggest shocks of my scientific life, as I realized immediately that Lynen was probably right. The Lynen intervention on Friday led to a frantic weekend of experimentation, resulting in a second letter to Nature,^5^ retracting the erroneous conclusion of the first one. It also reported, however, the discovery of the transamination pathway for glutamate oxidation, which turned out to be the predominant one for glutamate oxidation in mammalian tissues.^6^ It required an intra‐mitochondrial aspartate transaminase, glutamate oxaloacetate transaminase 2 [GOT2], which had already been found in Amsterdam and in two other labs (see Reference 7). In fact, the existence of this (apparently superfluous) GOT2 was one of my reasons to think of a MAS later. With hindsight our stupid mistake may seem incomprehensible, because the Krebs citric acid cycle with its substrate‐linked phosphorylation step had already been fully worked out and was in textbooks. It shows how persuasive generally held misconceptions can be, not only for students, but also for a seasoned and highly critical biochemist like Slater.

I went back to my main thesis project, the properties of tumor mitochondria and the pathways for the oxidation of cytosolic NADH^a^ by mitochondria.^1^ Al Lehninger had already shown in 1951^8^ that isolated mitochondria are unable to oxidize added NADH unless artificially swollen to allow the NADH to reach the mitochondrial Complex I. In my own experiments, I had found that the mitochondrial compartment does not exchange NADH or NAD with the cytosol in intact tumor cells.^9^ When cytosolic NAD was rapidly degraded by nuclear DNA damage,^b^ the mitochondrial NAD pool remained relatively untouched.^9^ That implied that the oxidation of cytosolic NADH by mitochondria required substrate shuttles, that is, substrates that are reduced in the cytosol and that can be oxidized by the mitochondria to yield a substrate that can return to the cytosol for reduction. The best documented of such shuttles was the glycerol‐3‐phosphate (glycerol‐P) cycle, independently discovered by Sacktor in the United States and Zebe and Bucher in Germany in insect flight muscle (see Reference 13). This cycle operates with a cytosolic glycerol‐P dehydrogenase and a mitochondrial analog located on the outside of the inner mitochondrial membrane, where it interacts with glycerol‐P in the cytosol and feeds reducing equivalents into the respiratory chain at ubiquinone. This cycle therefore neither requires transport of the glycerol‐P through the mitochondrial membrane, nor Complex I of the respiratory chain.

**FIGURE 1:**
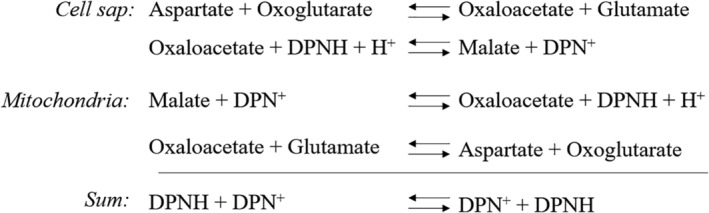
The MAS as presented in Reference 13 ^a^

My detailed study of Ehrlich ascites tumor cells in 1962 led to the conclusion that it is “improbable that any of the known or suggested pathways for the oxidation of extra‐mitochondrial NADH is operative in our strain of ascites‐tumor cells.”^1^ In search for an alternative, “as yet unknown pathway,” I came up with the MAS.^1^ This suggestion was worked out in more detail in two published contributions to symposia.^13^, ^14^ The initial MAS was just a series of equations. Colorful, persuasive pictures did not exist yet. In Slater's lab, we were told to balance our equations, however—what you put on the left of the arrows should fit what you put on the right—and this I did (see Reference 13), as shown in Figure 1.

There was an alternative to the MAS, the malate cycle, proposed by Bücher and Klingenberg.^15^, ^16^ This invoked the mitochondrial export of oxaloacetate in exchange for uptake of malate. I considered this alternative improbable, as the oxaloacetate concentration calculated from the equilibrium constant of the malate dehydrogenase (MDH) reaction was very low (see Reference 13) and because the mitochondrial membrane was (erroneously) thought at the time to be impermeable for oxaloacetate (see Reference 13). Moreover, the MAS explained the need for a highly active GOT2 in mitochondria, which was present in high concentration and in constant proportion to Krebs cycle enzymes in the tissues studied.^17^

It remained unclear, however, how the MAS could lead to oxidation of cytosolic NADH, as there were already indications in 1961 that the free NADH/NAD ratio was much higher in the mitochondria than in the cytosol. At the time, reliable data for the free NADH/NAD ratio in the cytosol were available from the work of Bücher and co‐workers (see References 15 and 16).^c^ They had shown that the reaction catalyzed by the highly active cytosolic lactate dehydrogenase (LDH) is in equilibrium in cells, just like the reaction catalyzed by cytosolic MDH. They deduced from their analyses of substrate couples, pyruvate/lactate and oxaloacetate/malate, that the free NADH/NAD ratio was much lower than the ratio of total NADH and NAD, presumably because most of the NADH is bound to dehydrogenases.

Similar data were not available for mitochondria, but following a suggestion of Klingenberg (see References 13 and 18), I used the couple beta‐hydroxybutyrate/acetoacetate to calculate the mitochondrial ratio of free NADH/NAD, using analyses published by Williamson and Krebs^19^ for rat hearts perfused with medium containing beta‐hydroxybutyrate. Strikingly, the betahydroxybutyrate/acetoacetate became constant after 30 min of perfusion,^19^ suggesting that the substrate couple might be in equilibrium. Since beta‐hydroxybutyrate dehydrogenase is exclusively located in mitochondria, the apparent equilibrium allowed me to calculate the free mitochondrial NADH/NAD ratio, using the known equilibrium constant of the dehydrogenase.^13^ The value found, 0.1, was indeed much higher than the NADH/NAD ratio published for the cytosol, which was 0.001. The general validity of this approach was later shown by the Krebs lab.^20^

**FIGURE 2:**
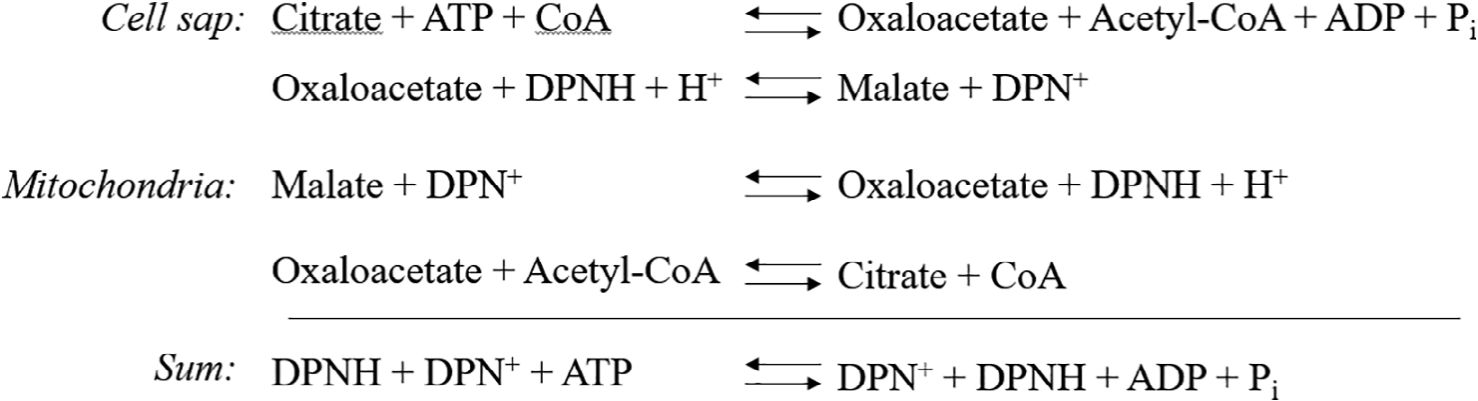
A possible energy‐expending reaction to drive the oxidation of cytosolic NADH. The malate‐citrate cycle as presented in Reference 13

The high probability that the NADH/NAD ratio was much higher in the mitochondria than in the cytosol, raised an obvious problem for the function of the MAS that I had proposed: oxidation of cytosolic NADH, as this would have to occur against the steep concentration gradient. To save the MAS from premature oblivion, I therefore proposed that the shuttle might be “coupled with an energy‐expending reaction,” driving it in the direction of cytosolic NADH oxidation. As an example, I made up the malate‐citrate cycle (Figure 2), in which the energy is generated by the cleavage of citrate and ATP in the citrate cleavage reaction.^13^ This paper biochemistry would come to life later, as discussed below in this article.

I had no time in 1961 to hunt for the postulated energy‐providing reaction driving the MAS, since I was back in the clinic, finishing my medical internships. It would last to the early 1970s until LaNoue and coworkers found that the efflux of aspartate from intact mitochondria is energy‐dependent and powered by the proton‐motive force over the mitochondrial inner membrane.^d,^ ^21^, ^22^, ^23^, ^24^

I never went back to cytosolic NADH oxidation in mammalian cells, but I did study the process later in African trypanosomes. In the bloodstream of their host, these uni‐cellular parasites rely on glycolysis, but with pyruvate as end product. The trypanosomes repress mitochondrial biogenesis in this life‐cycle stage. Hence, no respiratory chain and no MAS. Reoxidation of cytosolic NADH is exclusively taken care of by a glycerol‐P cycle. Disappointing MAS‐wise, but this work did lead to the discovery of a new organelle, the glycosome, in which the trypanosomatids pack most of their glycolytic enzymes.^25^

In the meantime, the MAS had become quite popular. The early follow‐up papers on the MAS still quoted my work as the Borst cycle, but my symposium papers were poorly accessible and eventually forgotten in the 1970s. In Amsterdam, however, the MAS is still chauvinistically taught to medical students as the Borst cycle by the current staff of the Biochemistry Department.

## 2 EARLY WORK ON THE MAS

The MAS made no splash in 1962. It lasted until 1965 before transaminations were again invoked for facilitating transport over the mitochondrial membrane by Lardy et al.,^26^ who studied gluconeogenesis from pyruvate in liver. This required reducing equivalents from the mitochondria and the most plausible source was malate. Although no dicarboxylate carrier had been identified yet in the mitochondrial membrane, it seemed unlikely that charged anions, such as malate or oxaloacetate, would readily diffuse through a membrane, and therefore an exchange process looked more plausible. Since oxaloacetate concentrations were known to be very low in the cell, aspartate was a more acceptable alternative. Lardy was not aware of my work, but in a later paper, he referred to Reference 13 and added that the MAS was a plausible route for getting reducing equivalents from mitochondria to cytosol, more plausible than the reverse reaction,^13^ which would require energy input.^27^ Plausible, but incorrect, as we now know. How reducing equivalents from the mitochondria are actually transferred to the cytosol, is discussed in Reference 28.

Acceptance of the MAS was probably stimulated by its adoption by the lab of Nobel laureate Hans Krebs.^e^ The Krebs lab determined the free NAD redox state in mitochondria and cytosol in rat liver under various conditions using the methodology developed in Bücher's lab, outlined and refined in Reference 13. The landmark Krebs' paper^20^ is still quoted today and may have alerted other investigators to my, somewhat obscure, early MAS papers. Hassinen^29^ then reconstructed the MAS in vitro with liver mitochondria and purified cytosolic components and his approach was followed by several other groups.^30^, ^31^, ^32^, ^33^, ^34^ This worked well, because energized respiring mitochondria were used, which were able to get reducing equivalents into mitochondria against a concentration gradient. The process was inhibited by butylmalonate, so it required the dicarboxylate translocator (see in the following), and by two inhibitors of transaminases, cycloserine^34^ and aminooxyacetate,^35^, ^36^ indicating that at least one transaminase was involved in the MAS. Cycloserine was later shown to poorly penetrate the mitochondrial membrane, indicating that inhibition of GOT1 was sufficient to inhibit the MAS (see extensive discussion of the early work on these inhibitors by Reference 28).

In 1970, Robinson and Halperin^33^ made the first attempt to quantitate MAS activity. They reconstructed the “Borst shuttle system”^33^ by adding a mix of enzymes and substrates to rat‐liver mitochondria and found rapid oxidation of the added NADH when aspartate was added. This method was used in several later papers, providing a more quantitative picture of the MAS.

At the time when I worked on the MAS, in 1961, there was little discussion in the lab about the mechanism of substrate uptake in mitochondria. Everybody knew that NADH was unable to penetrate intact mitochondria, but how the highly charged substrates used in our daily experiments managed to get through the mitochondrial inner membrane, was not an issue, as far as I remember. That changed in the 1960s and in a few years most of the mitochondrial substrate transporters were identified, mainly in the laboratories of Chappel, Meijer/Van Dam/Tager, and Klingenberg (reviewed in References 28, 37, and 38). The most relevant transporters for the MAS are members of the SLC25 mitochondrial transporter family (reviewed in Reference 39):

- The dicarboxylate translocator (SLC25A10), inhibited by butylmalonate. This translocator can exchange dicarboxylates, such as oxaloacetate, malate, and 2‐oxoglutarate, but inorganic phosphate (Pi) can also act as counter‐ion.
- The 2‐oxoglutarate translocator (SLC25A11), which also exchanges several dicarboxylates, but not against Pi.
- The glutamate–aspartate translocators (SLC25A12 and A13), usually known as the aspartate–glutamate carriers (AGCs); A12 is often referred to as citrin, A13 as aralar (reviewed in Reference 40).

These studies of the mitochondrial translocators added the malate‐2‐oxoglutarate exchange required for the MAS and also confirmed that oxaloacetate poorly gets through the mitochondrial inner membrane, because the relevant translocators, the dicarboxylate and the 2‐oxoglutarate translocators, have a low affinity for oxaloacetate (see Reference 41). That provided additional justification for the incorporation of the double transamination in the MAS.

It remained to be shown that the MAS is a major and indispensable route for the oxidation of cytosolic NADH in vivo. This was done by John Williamson and coworkers with perfused rat liver and heart, using metabolic labeling experiments, metabolite analyses, metabolic perturbations, and inhibitors.^22^, ^31^, ^42^, ^43^, ^44^, ^45^ I cannot do justice to this impressive series of thorough investigations and present only some examples. Rat liver contains a very active NAD‐linked xylitol dehydrogenase and with xylitol as substrate in the perfusion medium, the metabolism of xylitol is limited by the oxidation of cytosolic NADH. Using inhibitors, such as rotenone (inhibits the NADH dehydrogenase complex of the mitochondrial respiratory chain, Complex I), aminooxyacetate, and cyanide, the authors showed that both the MAS and the glycerol‐P cycle contribute to the oxidation of NADH and that the activity of these cycles was rate‐limiting if the respiratory chain was operating at full capacity. With glucose in the perfusion medium, the NADH generated in the glyceraldehyde‐3‐P dehydrogenase (GAPDH) reaction drives up the NADH/NAD ratio in the cytosol, raising the malate concentration and activating the malate‐2‐oxo‐glutarate exchange. The capacity of the MAS could account for the oxidation of cytosolic NADH, based on flux measurements of key metabolites and on the activity of a partially reconstituted MAS with isolated rat‐heart mitochondria.^22^, ^31^, ^42^, ^43^, ^45^, ^46^, ^47^ In the perfused rat heart, the glycerol‐P cycle is unimportant (see Reference 13). The glycerol‐P cycle can be induced by making the rats hyperthyroid, but even then, it remains a minor contributor.^47^ The MAS appears to be the major player in NADH oxidation.^47^

Later studies with perfused isolated rat‐liver cells showed that the respiratory chain is not always the rate‐limiting factor in the operation of the MAS. Meijer et al.^48^ studied ethanol oxidation by isolated perfused rat liver cells and found that the flux through the MAS can be kinetically limited by the low concentration of metabolites. Ethanol is oxidized in two steps: in the cytosol it is converted into acetaldehyde by NAD‐linked alcohol dehydrogenase. The acetaldehyde is oxidized by aldehyde dehydrogenase in the mitochondrial matrix. In liver cells from fed rats, the rate of ethanol oxidation was limited by the respiratory chain, which also needs to oxidize the NADH produced by acetaldehyde oxidation. In cells from starved rats, control shifted to the metabolic part of the MAS, because the availability of MAS metabolic intermediates became limiting. This was shown by the stimulation of ethanol oxidation by the addition of malate or other MAS metabolites to the liver cells.

A more recent study modeled the changes in MAS metabolites when hearts go anaerobic.^49^ The MAS stops the cytosolic NADH/NAD ratio and malate shoot up, whereas mitochondrial malate falls. Aspartate nearly disappears from both mitochondria and cytosol, a phenomenon, studied in detail later in cells without functional respiratory chain; these cells are dependent on exogenous aspartate (see in the following).

**FIGURE 3:**
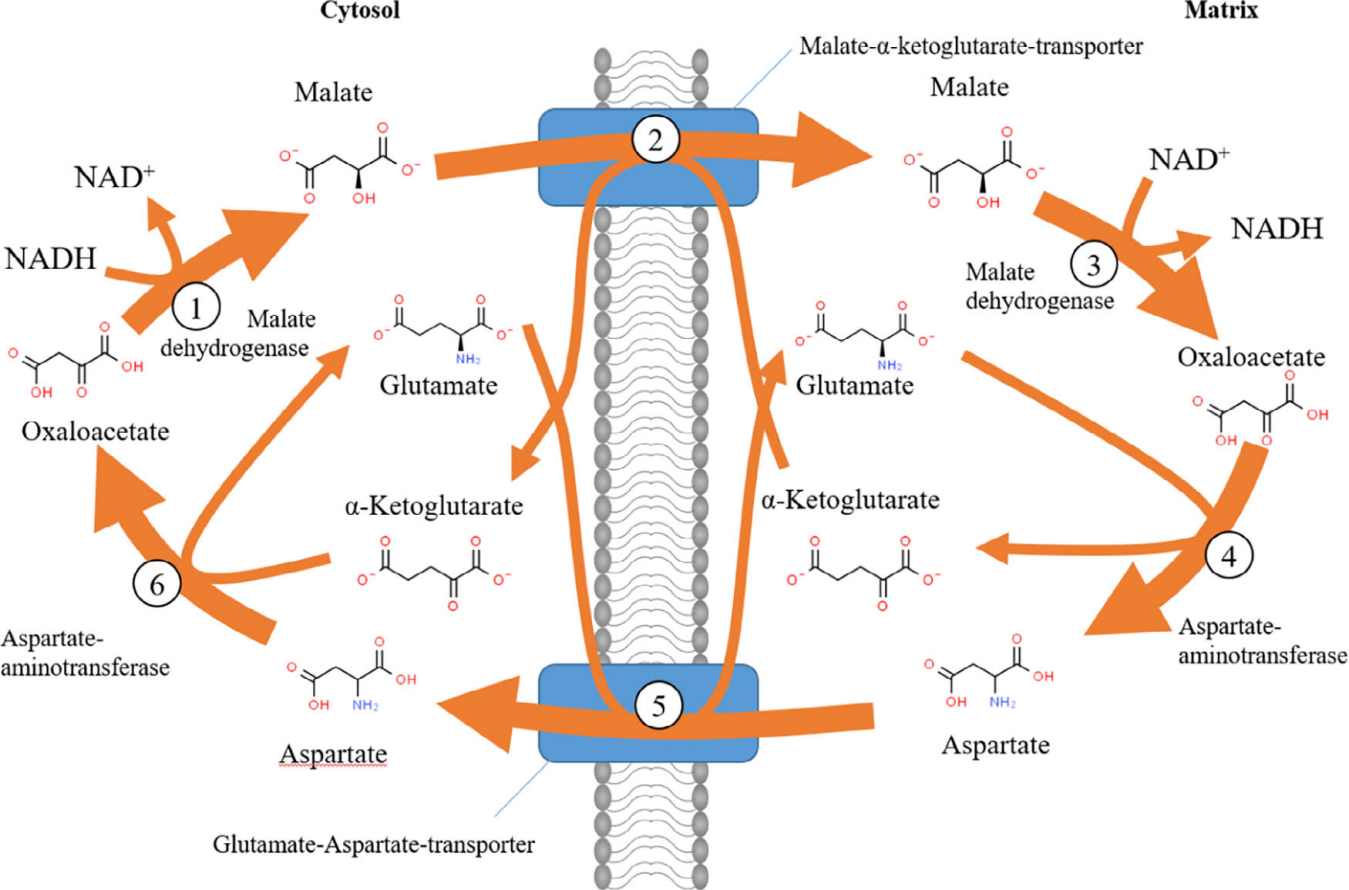
The malate–aspartate shuttle for transporting reducing equivalents from cytosolic NADH into the mitochondrial matrix (after figure 18–25 of Lehninger's Principles of Biochemistry^50^)

These experiments demonstrated a functioning MAS in intact rat tissues and isolated rat liver cells, but also provided the first indication for the energy‐requiring reaction driving the MAS in the direction of oxidation of cytosolic NADH: LaNoue and Williamson found that the export of aspartate from the mitochondria was blocked by uncoupling agents, suggesting that the export required energy.^22^, ^23^, ^31^ LaNoue and coworkers then elucidated the stoichiometry of the reaction catalyzed by the glutamate–aspartate translocators in landmark papers^21^, ^23^: for every aspartate molecule effluxed, mitochondria take up one glutamate molecule and one proton. Hence, the MAS is driven in the direction of cytosolic NADH oxidation by the proton‐motive force. The magnitude of the proton electrochemical gradient across the mitochondrial membrane of roughly 220 mV explains the 100‐fold difference between the NADH/NAD ratios in mitochondria and cytosol.^28^ This completed the picture of the MAS now in textbooks and shown in Figure 3.

**FIGURE 4:**
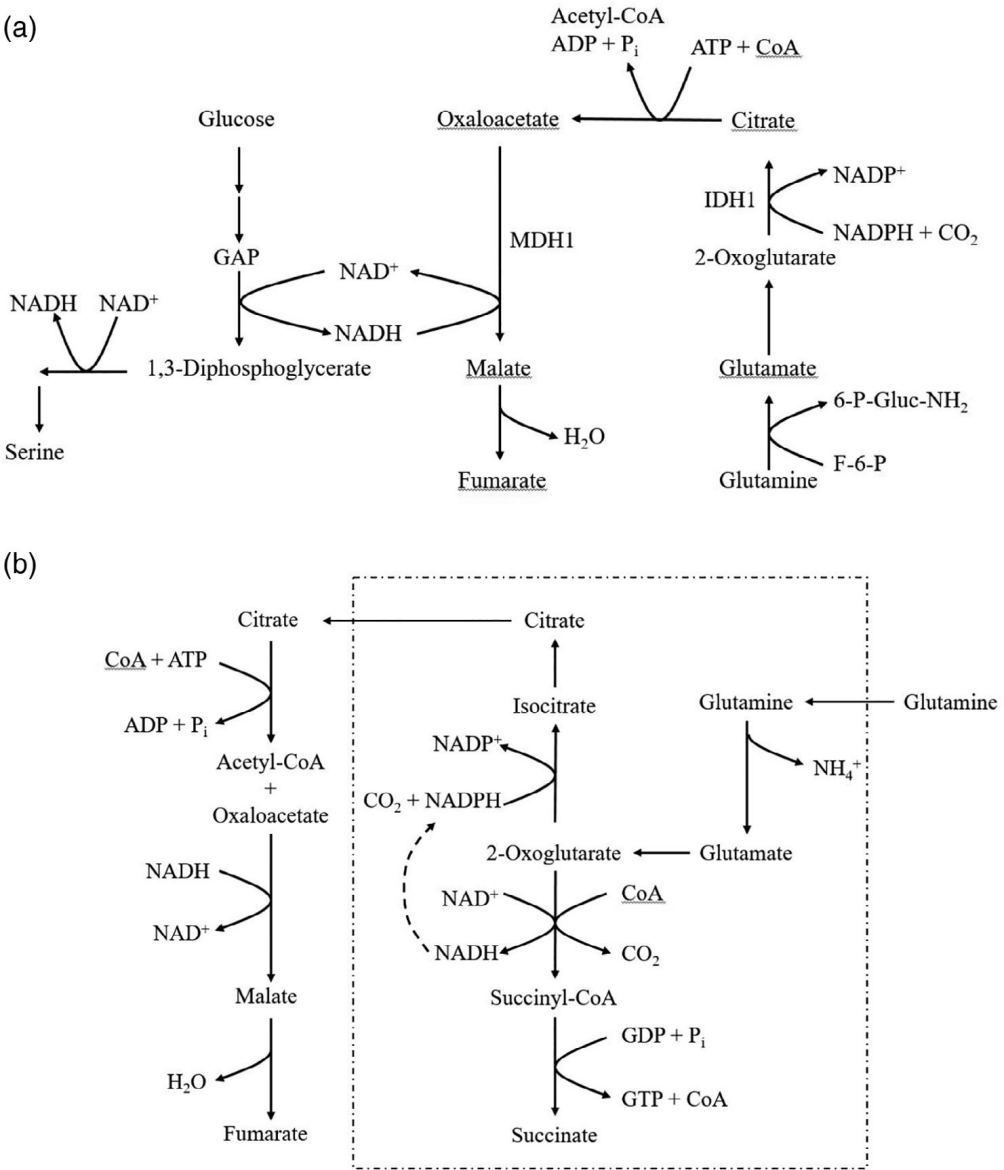
The two versions of reductive carboxylation that allow cells to circumvent the MAS (a). The cytosolic version, adapted from Reference 51. This picture is schematic; the malate and fumarate produced in the cytosol are in part exported from the cell. (b) The mitochondrial version, adapted from Reference 52. This picture is also schematic; the succinate produced must be transported from the mitochondria and the cell. The broken line indicates the mitochondrial membrane. 2‐OG, 2‐oxoglutarate; 6‐P‐Gluc‐NH2, glucosamine‐6‐P; Bis‐P‐Glyc, 1,2‐bis‐P‐Glycerate; F‐6‐P, fructose‐6‐P; GAP, glyceraldehyde‐3‐P; OAA, oxaloacetate; Pi, inorganic P

Although the MAS is usually shown to function in the oxidation of NADH produced by glycolysis, it should be obvious that glycolysis is not the only pathway reducing NAD in the cytosol. Oxidation of the glycolytic intermediate 3‐phosphoglycerate by the NAD‐linked P‐glycerate dehydrogenase is another important source of cytosolic NADH (see Figure 4a). This pathway is required for the synthesis of vital metabolites, such as serine and glycine.^53^ A defective MAS leads to lack of serine and a defect in one‐carbon metabolism and this is seen in the inborn MAS disease caused by defective GOT2,^54^ as discussed in the following. Another pathway adding to the generation of cytosolic NADH is the oxidation of fatty acids in peroxisomes. The oxidation of 3‐OH‐acyl‐CoA yields NADH, which is transferred to the cytosol by a substrate shuttle involving pyruvate/lactate and the peroxisomal and cytosolic LDH isoenzymes.^55^

Whether the MAS is a general feature of cells with functional mitochondria remains to be seen, as no systematic study has been published. There are certainly exceptions, such as insect flight muscle and white adipose tissue, which are very low in GOT2.^33^ However, the Bücher group already showed that GOT2 is a constant feature of mitochondria in the cells studied^17^ and the glutamate–aspartate translocator is found in all branches of eukaryotic life.^40^ Serving the MAS seems a reasonable function for both enzymes.

## 3 CONTROL OF MAS ACTIVITY

Obviously, the MAS will become rate‐limiting in the oxidation of cytosolic NADH when there is interference with any of the steps in the shuttle. This includes experiments with inhibitors of the transaminases (such as aminooxyacetate), of the dicarboxylate translocator (butylmalonate), of the respiratory chain (phenformin), of succinate dehydrogenase (3‐nitropropionic acid; Reference 56), or downregulation of components of the respiratory chain, or of the steps in the Krebs cycle that are required for the MAS (see Figure 3). Inborn errors in each of these steps duplicate some of the results of these inhibitor experiments, as will be discussed in the following.

As noted before, Lardy et al.^27^ invoked the MAS to deliver reducing equivalents to the cytosol for gluconeogenesis and some authors^57^ still conclude that “the MAS is reversible.” This may be correct in theory, but improbable in practice. As long as the mitochondria are not completely uncoupled, the proton motive force makes the aspartate–glutamate translocator in principle uni‐directional toward glutamate import (see References 21 and 28). The present consensus is that the MAS is irreversible.

Early work indicated that the aspartate–glutamate translocator could control flux through the MAS. LaNoue and Williamson^31^ reconstructed the (partial) MAS with isolated rat‐heart mitochondria and concluded that efflux of 2‐oxoglutarate and aspartate were rate‐limiting when the respiratory chain was maximally active. More recently the overexpression of the aspartate–glutamate translocator citrin was studied in intact cells. Increased expression of a transfected construct encoding citrin in non‐transformed mouse myoblasts raised the total cellular NAD/NADH ratio and the concentration of aspartate, indicating that in these cells, this transporter predominantly controls the flux through the MAS.^58^ This was extended by Rabinovich et al.,^59^ who overexpressed citrin in a melanoma cell line and found that this increases aerobic glycolysis and invasiveness in an in vitro assay.^f^ Both Alkan et al.^58^ and Rabinovich et al.^59^ notice that citrin is upregulated in some human tumors, but whether other MAS components were up was not checked. These results suggest that citrin may control the overall rate of the MAS and that upregulation of the MAS may promote glycolysis.

The interpretation of these results should take into account that the activity of the aspartate–glutamate translocators is 2–3‐fold stimulated by the Ca‐ion concentration^60^, ^61^ and that this stimulation is dependent on the nature of the translocator, aralar requiring higher concentrations of calcium for stimulation than citrin.^60^ Under some physiological conditions, MAS activity will therefore be affected by changes in the cytosolic Ca‐ion concentration. Leverve et al.^62^ found, for instance, that treatment of perifused hepatocytes with phenylephrine resulted in a stimulation of the mitochondrial efflux of aspartate, which they attributed to a rise in cytosolic Ca‐ions, (an interpretation preceding the actual discovery of the Ca stimulation of the aspartate–glutamate transporter.) It is possible that this calcium stimulation plays a physiological role in heart and brain^60^, ^63^ as well, in which cellular calcium concentrations may vary more than in other tissues, but in most tissues, these variations are considered not of major importance for MAS activity.^60^

Although most investigators think that the aspartate–glutamate translocator usually controls the flux through the MAS, a different conclusion was reached by Yang et al.^64^ GOT2 is acetylated on lysines and the level of acetylation is controlled by the mitochondrial NAD‐dependent deacetylase SIRT3. Yang et al.^64^ found that acetylation promotes the association of GOT2 with MDH2. Even though this association does not affect the activity of GOT2, the authors attribute a profound stimulation of the MAS to the teaming up of GOT2 and MDH2 and they think that this should have major consequences for the functioning of the HEK293 and pancreatic ductal carcinoma cells used in these studies. Since the 14 acetylated lysines are highly conserved in evolution, the authors conclude that the post‐translational control of the MAS uncovered by them could be a more general mechanism to control the MAS in eukaryotes.

Although the authors presented an impressive set of data to support their conclusions—indeed all perfectly fitting the picture sketched—I am not convinced by their results. They maintain that the association of GOT2 and MDH2 leads to substrate channeling and that this explains the low concentration of oxaloacetate in cells, but this low concentration could merely reflect the equilibrium constant of the MDH reaction, which is far in the direction of malate (see Reference 13). Although the concept of channeling is regularly invoked in biochemical texts, calculations usually show that diffusion times are so short in organelles that channeling does not have an effect.^g^ In the case of the GOT2‐MDH2 duet it should be noted that a complex has also been claimed to exist for the Krebs cycle enzymes and this complex includes MDH2.^67^ How MDH2 can be in two complexes simultaneously remained undiscussed.^64^

I find it also unfortunate that the authors do not address the evidence that there are other steps in the MAS that appear to be more important for the overall flux rate of the MAS (e.g., citrin) than GOT2 and MDH2, enzymes that are usually assumed to be close to equilibrium. This objection is reinforced by evidence that the acetylation of mitochondrial proteins is a non‐enzymatic process, dependent on the intra‐mitochondrial acetyl‐CoA concentration. Acetylation affects a large range of proteins, including GOT2 but also MDH2. Interestingly, the activity of MDH2 IS increased by acetylation, in contrast to that of GOT2.^68^ Since many mitochondrial proteins are acetylated, citrin and other translocators might be acetylated as well and potentially activated. This remains to be investigated.

Acetylation of MAS proteins was also invoked by Wang et al.^68^ for the control of the MAS. They stimulated fatty acid breakdown in pig liver raising the level of acetyl‐CoA and the degree of acetylation of mitochondrial proteins. They isolated mitochondria from these livers and tested the MAS activity in a reconstructed system as in Reference 33. After stimulation of fatty acid breakdown in the livers, the MAS activity went up three‐fold.^h^ This is impressive, but nevertheless I find it more likely that acetylation of citrin is responsible for this increase than the association of GOT2 and MDH2. Clearly more work should be done to study the putative post‐translational control of the MAS.

The observation that increasing key steps in the MAS can increase glycolysis seems in contradiction with modeling of metabolism, which indicates that glycolysis is regulated relatively independently of the Krebs cycle^69^ and that there is no aerobic glycolysis if the capacity of MAS and mitochondrial respiratory chain is sufficient to re‐oxidize the NADH generated in the GAPDH reaction.^70^ The possibility that glycolysis can be activated by increasing the MAS and lowering the cytosolic NADH/NAD seems also in contradiction with the conclusion of Tanner et al.^69^ that the rate of glycolysis in the cells they studied is NOT at the GAPDH reaction, which produces the NADH to be oxidized via the MAS. Also, Park et al.^71^ find that in human bronchial epithelial cells, the flux through glycolysis is controlled by the level of PFK. This level is affected by the E3 ubiquitin ligase TRIM21, which targets PFK for proteasomal degradation. Clearly more work is required to disentangle this discrepancy.

What limits the MAS features also in the ongoing discussions of the Warburg effect, the aerobic glycolysis that characterizes many (but not all) tumor. I mentioned already the upregulation of citrin in some tumors. Other tumors have increased levels of MDH and this is reported to correlate with poor prognosis of patients. Zhang et al^72^ studied Non‐small Lung Carcinomas and found a large increase in MDH activity. Both MDH1 and 2 were up, but only upregulation of MDH1 correlated significantly with poor patient survival. In a panel of tumor‐derived cell lines, knock‐down of MDH1 affected viability more severely than knock‐down of MDH2.^i^

A more detailed analysis of the role of MDH1 was made by Hanse et al.^73^ They used glucose labeled with deuterium at the 4 position. This deuterium will be transferred to NAD in the GAPDH reaction and from there to acceptors. The most highly labeled acceptor‐compound in HeLa cells was malate, even more than lactate. Some of the malate was excreted, like lactate, suggesting that malate was in part purely used as electron acceptor. The authors also studied the MDH1 KO Jurkat cell line constructed by Birsoy et al.^74^ and showed that that this line multiplies somewhat slower than the WT and that this is remedied by adding excess pyruvate as electron receptor. Unexpectedly, the MDH1 KO cells continue to proliferate even if an inhibitor of LDH was added. Apparently, the cells have access to a multiplicity of acceptors to get NADH oxidized. The authors went on to show that the cells metabolize glutamine at high rates and raise the possibility that reductive carboxylation could be used to oxidize cytosolic NADH (see in the following), although they did not investigate this. Whether these cells contain an active glycerol‐P cycle is not reported either.

The authors looked at MDH1 levels in human tumors as well. Strikingly, they found amplification of the MDH1 locus in 11% of the squamous cell lung cancer specimens, and they provide evidence that this is indeed due to selection for increased MDH1 activity.^73^ How increasing the MDH1 activity would benefit the tumor is not explained.

GOT1 has usually been considered to be in equilibrium, at least in rat liver (see Reference 13), but this has been challenged by a paper of Wang et al.^75^ on virus‐infected cells. They found that several viruses stimulate in their host cells the production of an lncRNA that traps GOT2 in the cytosol. They concluded that the additional GOT activity in the cytosol stimulates virus replication and they supported this interpretation with extensive evidence. Dragging GOT2 from its proper location in the mitochondria should also disrupt the MAS, however. The authors did not even mention this complication and they also did not measure whether GOT1 is sufficiently far from equilibrium in their virus‐infected cells to make it worthwhile to add a little GOT2 to the cytosolic GOT activity.^75^

Obviously, the capacity of the MAS is not only affected by the levels of the proteins involved, but also by the reaction kinetics that are determined by substrate availability, which may vary widely. The work of Meijer et al.^48^ on starved rat liver cells was already mentioned. The point is further illustrated by a study of a large set of cancer cell lines with mutations in phosphatidylinositol 3‐kinase (PI3K), which are known to drive oncogenic transformation. Oncogenic PI3K drives hyperactivity of glycolysis and Ilic et al.^76^ find that this makes the cell hypersensitive to inhibition of 2‐oxoglutarate dehydrogenase. The resulting increase in 2‐oxoglutarate concentration drives the aspartate transaminases toward formation of glutamate and oxaloacetate, lowers aspartate and increases the cytosolic NADH/NAD ratio. The authors concluded that the low aspartate levels inhibit the MAS and that the resulting inhibition of glycolysis interferes with cell multiplication. A complication in the interpretation of these experiments is that the multiplication defect was not reversed with excess pyruvate, which provides an electron acceptor, whereas it was reversed by excess aspartate. The excess aspartate also rectified the decreased NAD/NADH ratio in the cytosol, presumably because it also provided oxaloacetate. In cells with a blocked respiratory chain, as discussed in the following, aspartate has been shown to be critical for cell multiplication, because it is essential for purine and pyrimidine synthesis and other vital biochemical reactions. Usually glutamine in the medium can provide the aspartate required, but when 2‐oxoglutarate dehydrogenase is inhibited, the high 2‐oxoglutarate levels prevent this.^76^ Some other cancer cell lines that do not carry an oncogenic PI3K mutation are hypersensitive to inhibition of 2‐oxoglutarate dehydrogenase inhibition as well.^77^

In conclusion, there are various ways in which the MAS may be controlled, depending on the conditions. In highly aerobic tissues such as liver and heart, the MAS does not appear to limit the oxidation of cytosolic NADH, as long as sufficient levels of MAS metabolic intermediates are available. The overall flux seems to be controlled by the aspartate–glutamate translocator and possibly the 2‐oxoglutarate translocator. Under various conditions, individual MAS components may increase in activity by upregulation at the transcriptional level or by acetylation. How this benefits cell functioning is often unclear and needs more work.

## 4 THE MAS VERSUS COMPETING CYCLES

The MAS is not the only route for oxidation of cytosolic NADH. The glycerol‐P cycle is the oldest, but even in 1962, there were several others, some of which already recognized as artifacts.^13^ In 1979, Dawson^78^ could list seven shuttles with some respectability in the only (brief) review ever dedicated to the MAS. Besides the MAS, the glycerol‐P cycle, and the disqualified malate‐oxaloacetate cycle, these included the malate‐citrate, the fatty acid, the pyruvate‐lactate and the branched alpha‐hydroxy acid amino acid shuttles. The pyruvate‐lactate shuttle is only used to transport reducing equivalents out of peroxisomes (see Reference 55). With the exception of the malate‐citrate cycle, there is no evidence that any of the other cycles operates in vivo^28^ and I am disregarding them here.

Initially, the glycerol‐P cycle was considered dispensable, as mice without the cytosolic GPD were healthy and just a little leaner.^79^ The double KO of the cytosolic and the mitochondrial branch of the cycle is embryonic lethal, however.^80^ This appears to be more due to the essential nature of the mitochondrial GP dehydrogenase than of the cycle.^80^ What determines the balance between MAS and glycerol‐P cycle is not well known. In hyperthyroid rats, the cycle is induced in the liver, but its contribution to oxidation of cytosolic NADH remains minor relative to that of the MAS (see Reference 28). There are indications that induction of the cycle is also possible when the MAS is compromised, as in inborn errors in the MAS (see in the following), but this remains to be studied more systematically, as in most studies the cycle is ignored.

More recently, the attention has shifted to the reductive carboxylation and the malate‐citrate shuttle (Figure 2) as a mechanism to circumvent the need for the MAS, when it is not available. This is discussed in a separate section in the following.

An unusual complication is the discovery of LDH associated with mitochondria. A recent prominent paper from the lab of DeBernardinis, one of the experts on tumor metabolism, finds that human lung tumors prefer lactate over glucose and that the label from glucose and lactate do not completely mix in the pyruvate pool.^81^ The authors raise the possibility that this lack of mixing is due to the entry of lactate into the mitochondria, where a mito‐LDH produces the pyruvate locally and delivers the NADH directly to Complex I. No shuttle required. If true, this would undercut a lot of work on shuttles. However, I find the evidence, even though extensive and often published in top journals,^82^ unconvincing. A crucial question is whether the mito‐LDH is in the matrix space (and in direct contact with the respiratory chain) or in the mitochondrial inter‐membrane space (where NADH requires a shuttle to deliver its reducing equivalents to the respiratory chain). The literature does not provide a clear answer. Even one of the key proponents of mito‐LDH, Brooks, initially had the enzyme in the matrix^83^ and now outside,^84^ showing that this field promotes imaginative interpretations. I have not found a paper showing the existence of an LDH with a mitochondrial targeting sequence. Without that sequence enzymes will not get into the mitochondrial matrix. I have not seen a single paper with a classical De Duve plot,^j^ quantitating the percentage of LDH that ends up in the mitochondrial fraction and the degree of contamination of this fraction with cytosolic enzymes. A recent paper^86^ that does make an attempt to analyze contamination concludes that LDH is only a contaminant of the murine heart muscle mitochondria where other investigators have found it (and continue to find it, see Reference 87). A complication disregarded by all mito‐LDH afficionados is the presence of LDH in peroxisomes (see Reference 55). This has also long been received with some scepticism, but a recent paper shows that there is some read‐through of the gene for LDH sub‐unit B, which results in a version of LDHB with a C‐terminal peroxisomal targeting sequence ending up in peroxisomes.^88^ This looks to me like a plausible explanation for LDH found associated with the cellular particle fraction. Hence, I concur with the sceptics and disregard matrix mito‐LDH until proven wrong by rigorous experiments.

## 5 LIFE WITHOUT MAS

Tumors often need to cope with adverse conditions. Oxygen can become limiting, reducing the capacity of the MAS. Tumors may also contain defects in the Krebs cycle, or even in components of the respiratory chain.^89^ This has led to an intense scrutiny of the metabolism in these tumors and the pathway for the oxidation of cytosolic NADH, as the blocks in the Krebs cycle also interrupt the mitochondrial part of the MAS at succinate dehydrogenase or fumarate hydratase. These defects decrease mitochondrial ATP production and force cells to rely more on glycolysis without MAS.

That mammalian cells can survive with mitochondria without a functional respiratory chain was already shown in the classical paper of King and Attardi.^90^ They produced a human cell line without any mitochondrial DNA and therefore without mitochondrial ATP production. These cells proliferated fine, provided they were supplied with exogenous uridine and excess pyruvate. The uridine is required to make pyrimidine nucleotides, because the mitochondrial dihydroorotate dehydrogenase is inactive if it cannot hook up to a functional respiratory chain. The mutant cells require the pyruvate as a sink for the oxidation of the NADH produced in the GAPDH reaction.^k^ This article has been followed more recently by a large volume of papers analyzing (tumor) cells with various (induced) defects in the mitochondrial respiratory chain. This line of research has received an impetus from studies providing suggestive evidence that patients treated with phenformin/metformin, an oral anti‐diabetic drug that inhibits the mitochondrial Complex I, have a decreased risk of getting cancer (see Reference 91). In the presence of metformin, cells require an electron sink, such as high pyruvate in the medium, similar to cells with a defect in the respiratory chain,^90^ but a variety of other acceptors is also effective. Sullivan et al.^92^ showed that alpha‐ketobutyrate can substitute for pyruvate. As reduction of the alpha‐ketobutyrate produces hydroxybutyrate, which is not metabolized and excreted, this proved that oxidation of cytosolic NADH is the only requirement (besides uridine) to allow cells to thrive without functional respiratory chain. This conclusion was underlined by experiments using NADH oxidases. The proliferation of human colon cancer cells inhibited at Complex I with metformin could be rescued by introducing a yeast NADH oxidase,^93^ which hooks up to the respiratory chain. Similarly, the proliferation of HeLa cells, in which the respiratory chain was blocked with inhibitors, could be rescued by the introduction of a bacterial NADH oxidase, both in the mitochondria and in the cytosol. Both constructs lowered the cytosolic NADH/NAD ratio.^94^

Sullivan et al.^92^ investigated the metabolic processes that limited cell proliferation if the respiratory chain was blocked and no electron acceptor was added. This turned out to be a single compound, aspartate.^92^ Exogenous aspartate will allow the cells to proliferate in the absence of electron acceptors and they conclude that the primary role of respiration is to produce aspartate. The essential role of aspartate was independently found by Birsoy et al.^74^, ^93^ They screened Jurkat cell lines for genes that are essential if Complex I of the respiratory chain is inhibited with phenformin. Their top hit was GOT1 and this enzyme was also essential in other cell types if the respiratory chain was inhibited. No other component of the MAS turned up in this screen. The GOT1 was found to be required to produce aspartate; the cells also required high pyruvate to drive down the cytosolic NADH/NAD ratio and allow MDH1 to produce sufficient oxaloacetate for GOT1 to make aspartate, as the equilibrium of the reaction catalyzed by MDH1 is very far toward malate (see Reference 15). In the absence of GOT1, pyruvate was unable to rescue proliferation of cells with a blocked respiratory chain. These cells are probably hypersensitive to DNA damage, as an inhibitor of poly‐ADPribose polymerase (PARP) activity, an enzyme using up NAD in DNA repair, mitigates the negative effect of metformin on cell multiplication.^93^

## 6 REDUCTIVE CARBOXYLATION TO CIRCUMVENT THE MAS

When the MAS becomes limiting tumors may use glutamine taken as an alternative substrate to accept reducing equivalents and to make aspartate.^95^, ^96^ This requires the reductive conversion of 2‐oxo‐glutarate in citrate, a process discovered in 1948 by Severo Ochoa.^97^ ^l^ There are two versions of this reductive carboxylation, a mitochondrial and a cytosolic one, as illustrated in Figure 4.

The literature on the topic is voluminous, but I discuss here only a few key papers:

- Metallo et al.^95^ studied several cell lines under hypoxic conditions. These lines compensated the decreased availability of the MAS by a cytosolic reductive carboxylation (Figure 4a), requiring uptake of glutamine, which is deamidated by glutamine‐fructose amidotransferase (see Reference 99), as there is no glutaminase in the cytosol.^m^ The glutamate produced is then transaminated to yield 2‐oxoglutarate, which is reductively carboxylated by nicotinamide adenine dinucleotide phosphate (NADP)‐linked isocitrate dehydrogenase 1 (IDH1). The citrate produced is split by ATP‐citrate lyase to yield the oxaloacetate needed to oxidize cytosolic NADH (cf. Figure 2). This work was extended by Gaude et al.^51^ who studied human osteosarcoma cell lines with up to 80% defective mitochondrial DNA. They also showed that the malate produced (Figure 4a) is partially converted into fumarate and both are excreted from the cell.
- Mullen et al.^52^ used two different cell lines, one with a block in the respiratory chain at cytochrome b, the other with a Krebs cycle block at fumarate hydratase. In the defective cell lines, glutamine taken up by the cells enters the mitochondria and is converted into 2‐oxoglutarate by a glutaminase and transamination. Part of the 2‐oxoglutarate is oxidized and another part is reduced by NADPH‐dependent IDH3 to form citrate, which is exported to the cytosol and split into acetyl‐CoA and oxaloacetate. The oxaloacetate is used to oxidize cytosolic NADH and to form aspartate by transamination. The aspartate production for use in the cytosol requires the mitochondrial AGC.^52^ The oxidation of 2‐oxoglutarate yields the NADH required for driving the reductive carboxylation and leads to succinate accumulation. The fate of succinate, which acts as the ultimate redox sink to allow reoxidation of NADH generated in glycolysis, was not specified.^52^ Presumably succinate is excreted.

These alternatives for the MAS are both not real cycles as the products are excreted from the cell. Why the cells with mitochondrial defects use either cytosolic or mitochondrial reductive carboxylation, is not clear. Metallo et al.^95^ mention that a contribution of the mitochondrial NADP‐linked IDH3 pathway is “probable,” but do not measure this. Gaude et al.^51^ suggest that the difference in pathway may be due to the degree of mitochondrial dysfunction, less complete in their case than in the cell lines of Mullen et al.^52^ Why some remaining MAS would promote exclusively the cytosolic rather than the mitochondrial pathway is not explained. In cell lines with a block in the Krebs cycle,^51^ mitochondria can still oxidize 2‐oxoglutarate and this may generate the reducing equivalents to drive the mitochondrial reductive carboxylation. Leonardi et al.^103^ conclude that “both IDH1 and IDH2 function interchangeably supports reductive carboxylation,” but it is unclear from their article where this is based on. They show that the oncogenic IDH mutations inactivate the reductive carboxylation function, but only of the mutated allele in the heterodimer. Even when IDH1 is mutated, there is 50% of WT IDH1 activity left. The possibility that the relative levels of the mitochondrial glutaminases versus the cytosolic glutamine‐fructose amidotransferase play a role is not discussed.

The reductive carboxylation pathway is dependent on GOT1, but aspartate can also be taken up from the medium. This requires the presence of a glutamate–aspartate transporter in the plasma membrane,^104^ unless high concentrations of aspartate are added to the medium.

Another link between cancer and MAS came from the finding that cancer cells that are dependent on the MAS are highly sensitive to knock‐down of the mitochondrial 2‐oxoglutarate dehydrogenase. Remarkably, some tumor cells with an alternative source of aspartate can do without any 2‐oxoglutarate dehydrogenase activity at all,^77^ even though accumulation of 2‐oxoglutarate can imitate the suppressing effects of WT p53.^105^

Although these profound defects in tumor cell lines are not representative of the real world in most growing tumors, there is no doubt that tumors often cope with an inadequate oxygen supply and hence with poorly contributing mitochondria. Alternatives to the MAS and a fully functional respiratory chain are then of physiological importance.^100^, ^101^, ^106^, ^107^

## 7 MORE ON THE MAS IN TUMORS

The MAS was discovered in Ehrlich ascites tumor cells.^1^ In 1972, Kovacević restudied these cells and concluded that the pathway could not operate, because the concentrations of aspartate were insufficient for the shuttle to work.^108^ This negative conclusion was based on rather indirect experiments and Kovacević did not find an alternative route for the oxidation of cytosolic NADH in his version of the Ehrlich tumor cells.^108^ Notably, a functional glycerol‐P cycle was also undetectable. In 1976, Greenhouse and Lehninger studied a larger set of tumors.^109^ Their conclusions were unambiguous: in all tumors, including two strains of the Ehrlich ascites cell tumor, the MAS was the main, or the only pathway for cytosolic NADH oxidation. They used several methods to study the MAS: they checked the conversion of added lactate into pyruvate and showed that this was completely inhibited by the transaminase inhibitor aminooxyacetate, as well as by rotenone which blocks the MAS, but not the glycerol‐P cycle, an approach introduced by Dionisi et al.^110^ Greenhouse and Lehninger used the method of Robinson and Halperin^33^ and the fate of labeled glucose to quantitate the MAS in their tumor cell line, and showed that the rates were adequate to mediate the oxidation of NADH derived from glycolysis or lactate oxidation.^111^ A complication of studies with the Ehrlich cells is that the original line, isolated in 1903, has been propagated since in several laboratories, resulting in sub‐lines that differ in properties. Some do have an operational glycerol‐P cycle, others do not (see discussion of older literature in Reference 13), but properties may even vary over time.^112^ Hence, the variation in results obtained with “Ehrlich ascites cells.”^108^, ^110^, ^112^, ^113^, ^114^

The early work on tumor metabolism was often driven by the quest for an explanation of the aerobic glycolysis of tumors, the Warburg effect,^115^ and the ability of high rates of glycolysis to suppress mitochondrial respiration, the Crabtree effect,^116^ ^n^ even though not all tumors display a high aerobic glycolysis and some normal tissues have substantial aerobic glycolysis as well (see References 117 and 118). In fact, I started my PhD research with the mission to find the factor that was responsible for the uncoupling of mitochondria in Ehrlich ascites cells, postulated with great authority by Warburg.^115^ ^o^ The conclusion of my thesis (in Dutch) was that increased aerobic glycolysis is an adaptation of rapidly growing tumors to limiting oxygen. The current views are in line with this (see References 120 and 121), although there is also more emphasis on the advantages of ample glycolysis for the production of more biomass (see References 106 and 107). The MAS does not appear to play a role either in the Warburg or in the Crabtree effect^122^ and most tumors operate a perfectly normal MAS. Lack of capacity to oxidize cytosolic NADH is not the cause of aerobic glycolysis. In general, attempts to find a generalizable defect in tumor metabolism to replace the aerobic glycolysis proposed by Warburg have completely failed. A very recent review concludes that “The emerging view of cancer metabolism is that it is flexible and context‐specific, with few fixed broadly applicable liabilities.”^123^ Even that seems to me an optimistic summary of the field.

## 8 INBORN ERRORS IN THE MAS

A new protein or metabolic pathway in human tissues is followed like a shadow by a new inborn error. The MAS is no exception to this iron rule, but it took until 1987 before the lab of George Radda^124^ reported the first example of an inborn defect in the MAS. Only one patient was analyzed and this man had only exercise‐induced muscle pain. Since glycolysis seemed normal and isolated mitochondria had no detectable defect, the authors measured MAS activity in vitro with the method described in Reference 33 and found only 20% of the activity of normal control muscle mitochondria. The authors^124^ were unable to pinpoint the exact defect. The restriction of the abnormalities to skeletal muscle does not fit what we now know about the function of the MAS. With hindsight I doubt whether the defect in this patient with muscle disease was really in the MAS.

The first unambiguous MAS defect reported was the AGC mutant (AGC2, citrin, SLC25A13) found by Kobayashi et al.^125^ Although initially the link with the MAS was not made, the Saheki lab later worked out the consequences of this defect.^126^ Citrin is present at high levels in the liver and its absence results in neonatal intrahepatic cholestasis and adult‐onset type II citrullinemia. Both defects are explainable by the lack of aspartate in the cytosol. Aspartate is required for urea synthesis and a block in the export of aspartate from the mitochondria is not sufficiently compensated by uptake of aspartate from blood or conversion of asparagine into aspartate. Interestingly, these patients, although ignorant of the underlying biochemistry, tend to avoid carbohydrates and alcohol (which requires for its oxidation a functional MAS), and prefer foods rich in aspartate/ asparagine. The patients develop a fatty liver, which is attributed to the operation of the malate‐citrate shuttle (Figure 2). This shuttle transfers acetyl‐CoA from mitochondria to cytosol, where it promotes fatty acid synthesis.^126^ Early attempts to make a mouse model of the citrin defect failed, because the glycerol‐P cycle is far more active in mouse than in human liver. A double KO of both pathways reproduced the human phenotype of citrin deficiency, however.^80^ Initially it was thought that citrin deficiency only occurs in Asia, but it is now known to be a pan‐ethnic disease (as reviewed in Reference 127).

Whereas Palmieri^127^ had only one MAS defect to offer in his comprehensive review of mitochondrial transporter defects in 2008, soon more would follow. In 2009, Wibom et al.^128^ reported a Swedish patient with an inborn defect in AGC1, the aspartate–glutamate translocator isoform mainly active in brain and muscle. The patient had a severe neurodevelopmental disorder, but no elevated blood lactate, indications of mitochondrial dysfunction, or muscle defects. However, the glycerol‐P concentration in blood was elevated and the authors speculate that the glycerol‐P cycle may compensate in part for the absence of the MAS in brain and muscle. The dominant symptom in the patient was hypomyelination of regions in the brain and the authors attribute this to the blocked efflux of aspartate from the mitochondria, leading to a severe decrease in the formation of acetyl‐aspartate, which is essential for myelination. The disease is phenocopied by a mouse KO.^128^ A second (American) patient with identical symptoms was reported by Falk et al.^129^ They do find an elevated lactate level in the brain, however, and speculate that an energy‐deficient state and cytosolic glutamate accumulation may contribute to the disease symptoms.

In 2019, two additional MAS errors were reported, in GOT2^54^ and in MDH1.^130^ The GOT2 defect was found in three different families and was only partial. The complete absence of functional GOT2 is probably not compatible with life, as the corresponding KO mouse is an embryonic lethal.^54^ A HEK293 GOT2 KO cell line could be grown in Dulbecco's modified Eagle medium, but was severely deficient in serine and glycine synthesis. This deficiency was completely abolished by growing the cells in high concentrations of pyruvate as acceptor of reducing equivalents. This is very similar to the results obtained with tumor cells with a defective MAS, mentioned in the preceding sections. The GOT2‐deficient patients had an encephalopathy with epileptic seizures, like the AGC1‐deficient patients, but in addition, elevated blood lactate and ammonia. The seizures could be fully controlled by the administration of a combination of pyridoxine and serine. The pyridoxine was thought to stabilize the abnormal GOT2 as the enzyme needs pyridoxal‐5‐phosphate as an obligatory co‐factor, common to all transaminases. Serine production in the patients' cells was compromised by a high NADH/NAD ratio, inhibiting the P‐glycerate dehydrogenase reaction required for serine production (Figure 4a). The authors also advise to reduce carbohydrates and supplement the diet with fat and ketone bodies, which do not produce NADH in the cytosol.

The MDH1 defect was found in two patients from a single Arabian, consanguineous family.^130^ The patients had a global developmental delay, epilepsy and progressive microcephaly and severely diminished MDH1 activity. The most prominent biochemical alterations were elevated blood glutamate and glycerol‐P, the latter attributed to compensatory upregulation of the glycerol‐P cycle. The metabolic phenotype was only partially reproduced in the HEK293 MDH1 KO produced, which had only a limited elevation in glycerol‐P levels, but in addition, increased levels of aspartate and fumarate, all readily explainable by the metabolic defect. The difference between patients and HEK cells may be due to the residual MDH1 activity in the patients and/or metabolic adaptation. A mouse MDH1 KO has not been reported, but can also be expected to be embryonic lethal, like the GOT2 KO.

No inborn defect has yet been reported in the 2‐oxoglutarate translocator, required for the malate‐2‐oxoglutarate exchange in the MAS. It is possible that this defect can be compensated by a hyperactive dicarboxylate translocator, or that the defect is lethal. I have not seen attempts to disrupt the gene for this translocator in mice or cells. An inborn error in GOT1 also remains to be reported. Even though this can be expected to be an essential enzyme, there might still be partial defects, or alleles encoding an unstable enzyme lurking in the human population.

The MAS shares part of its intra‐mitochondrial path with the Krebs citric acid cycle. One would therefore expect disturbances in the MAS, caused by defects in succinate dehydrogenase, fumarase, and MDH2. These have been found,^89^ most recently a defect in MDH2.^131^ In these inborn errors, the disturbance of the Krebs cycle seems to predominate, however, and I am therefore leaving this section of the MAS out of this review. A more extensive description of the inborn errors in the MAS can be found in van Karnebeek et al. (to be submitted).

Defects in the MAS share with many other mitochondrial defects a high cytosolic NADH/NAD ratio.^132^ An ingenious approach to tackle this altered redox situation in patients with mitochondrial defects in vivo was recently reported by the group of Vamsi Mootha.^133^ They constructed a fusion protein of a bacterial lactate oxidase and catalase, LOXCAT, able to convert lactate and oxygen into pyruvate and water. When added to the medium of cultured cells, this enzyme was able to lower the lactate/pyruvate ratio in the medium and hence also intra‐cellularly. LOXCAT decreased the cellular NADH/NAD ratio in cells with a defective respiratory chain, upregulated glycolysis and restored cellular proliferation. Injected LOXCAT even decreased the NADH/NAD ratio in the heart and brain of mice treated with high doses of metformin. This is an original and gratifying way of reducing the need for an active MAS. It also uses the fact that the LDH reaction is in equilibrium and that alteration of the lactate/pyruvate ratio results in a corresponding change in the cytosolic NADH/NAD ratio, as first discovered in the Bücher lab more than 60 years ago^15^ and often forgotten. Obviously, Reference 133 only provides a proof of principle. LOXCAT is undoubtedly immunogenic and ways must be found to shield the enzyme from the host immune system.

## 9 CONCLUDING REMARKS

Nearly 60 years after the MAS started its biochemical career, it is still going strong. It has not only become an essential feature of biochemical textbooks, but it is also still the subject of intensive research. Many aspects of the MAS remain poorly understood, as illustrated in several sections of this review. There is more to come.

## ENDNOTES

a In the older papers quoted here NAD is still called DPN.

b In 1963, we still had no idea why DNA damage results in massive NAD degradation and most of the published explanations of the time were wrong.^10^, ^11^ It would last until 1979 to make the link with PARP using up NAD in DNA repair.^12^

c Theodor Bücher was one of the dominant figures of post‐War German biochemistry. A pupil of Otto Warburg and a typical, old‐style German professor himself, he did not suffer fools in silence and his persistent and forceful critique was feared by colleagues who had not done their homework properly. Although Bücher was not fluent in English, he realized that world leadership in biochemistry had shifted from, once dominant, Germany to the Anglo‐Saxon world. He drove the transformation of the old and respected Biochemische Zeitschrift into the Eur. J. Biochem. and he sent his best students for a postdoc to the United States. Bücher was not only an excellent enzymologist, but he also had a knack for technology. He was behind the development of famous instruments, such as the Eppendorf UV photometer and (Marburg) micropipette. His student Martin Klingenberg learnt to operate the novel double‐beam spectrophotometer in the lab of Britton Chance in the United States, and on his return was induced by Bücher to build the first European version. This was copied by Slater and his biophysicist Van der Tweel in Amsterdam. The instrument became known as the “Advantage Wheel” by a typing error. In the regular correspondence between Chance and Slater, Chance had mentioned the Van der Tweel instrument. Since Chance dictated his letters and did not correct the dictate, his puzzled secretary had turned Vandertweel into Advantage Wheel, to the joy of Slater who ran through the lab to share Chance's letter. It was Bucher who pushed my invitation to the prestigious, small Rottach‐Egern Conference.^13^ I probably caught Bücher's eye by an earlier talk in which I incorporated the Bücher famous concept of redox equilibrium. In painstaking analyses Bucher had shown that the reactions catalyzed by the main cytosolic dehydrogenases, lactate, malate, and G‐1‐P dehydrogenases are in equilibrium in several tissues. It is therefore possible to calculate the “free” NADH/NAD ratio in the cytosol from the substrate ratio's lactate/pyruvate, malate/oxaloacetate and dihydroxyacetone‐P.^15^ This concept was not immediately understood/accepted around 1960 (and even in 2020) and Bücher was obviously pleased to see it adopted in a talk by me, a mere medical student. It may have helped that I played the cello suites of Bach (not at my talk), because Bücher was a Bach fanatic. He had all Bach cantata's on record, a major investment at the time. Speaking at the Rottach‐Egern conference was a daunting prospect and I must have spent considerable time in preparing the talk in the weekends during my medical internships. The effort still shows.^13^

d This historical paper on the MAS was written at the same time as a review on inborn errors in the MAS, to which I contributed part of the text (Van Karnebeek et al., to be submitted). I did not succeed in avoiding all duplication.

e I had the pleasure of discussing my experiments with Krebs in 1959, when I had just scooped his lab by finding the transamination pathway for glutamate oxidation. He was not resentful and took time to discuss my experiments in detail, even though I was just a beginning graduate student. He seemed completely untouched by Krebs–cycle fame, a modest, congenial and decent man.

f Unfortunately, the authors only studied a single transfected cell line and not all the expected metabolic changes associated with increased MAS activity were found in the cells. Although the cytosolic NAD concentration (measured with a fluorescent probe) more than doubled by the citrin overexpression, the increase in the NAD/NADH ratio was not significant, because of the wide spread in the data. As this parameter was measured by the pyruvate/lactate ratio, the negative result could be due to the lack of equilibrium of the LDH reaction, which can occur if the rate of glycolysis is high.

g My scepticism toward channeling is due to the glycosome, the peroxisome‐like organelle in African trypanosomes containing most of the glycolytic system.^25^ Why would trypanosomes organize their glycolytic system so differently from other eukaryotes? An obvious answer at the time seemed channeling. The rate of glycolysis in bloodstream trypanosomes in very high and packing the enzymes close together would make the system more efficient, we thought. This proved one of the rare cases where the channeling model could be critically tested, because Michels and Opperdoes and their co‐workers determined the data required and then the group of Hans Westerhof modeled the glycosome. The result was conclusive: no channeling.^65^ Since that time I have been more skeptical of claims that enzymes associate to allow channeling. Proving that channeling plays a role is not easy. The fact that enzymes are packed together or associate does not suffice. More on this complex issue in a recent review.^66^

h It may seem puzzling that the MAS would need to go up so much in livers oxidizing fatty acids, which deliver their reducing equivalents in the mitochondrial matrix rather than in the cytosol, but the MAS is required to deliver aspartate to the cytosol for gluconeogenesis and malate for ureogenesis (see Reference 28).

i The authors suggest that MDH1 may have additional functions than contributing to the MAS, also because, somewhat unexpectedly, the increased MDH1 in tumor cell lines did not appear to affect the NAD/NADH ratio in the cells. Unfortunately, this analysis was limited to total cellular NAD and NADH and no attempt was made to measure the free cytosolic NAD/NADH ratio from the pyruvate/lactate ratio.

j The late Christian De Duve was not only a highly competent biochemist, but also a brilliant speaker. Being our Belgian neighbor and close by, he was invited regularly to Amsterdam, by the clinicians to speak about inborn errors in carbohydrate metabolism, and by the biochemists to speak about the new organelles he had discovered, lysosomes and peroxisomes. I was therefore exposed at an early stage to the rigorous methods he developed for the separation and characterization of cellular particulate fractions and to the dangers of just looking at a “purified mitochondrial” fraction. Unfortunately, the principles established by De Duve somehow disappeared from lab protocols and I am regularly amazed by the nonsense published in this area by reputable labs (see correspondence after Reference 85).

^k^ Biochemists often worry about the relative inefficiency of glycolysis in producing ATP compared with oxidative phosphorylation. Although this sentiment still permeates textbooks, it is unfounded. If your host provides ample sugar, glycolysis suffices for ATP production. Yeast and bloodstream trypanosomes do fine without any oxidative phosphorylation. They do need an electron acceptor, however, to allow glycolysis to generate carbon for cell multiplication and turn‐over. If every glucose molecule is completely converted into two lactate, there is ATP formation but no source of carbon. The electron acceptor allows part of glycolysis to operate without lactate formation and provide metabolites for growth.

l I spent 1963–1964 in Ochoa's lab in New York, NY, where I worked (with fellow post‐doc Charles Weissmann) on the replication of RNA bacteriophages in *E. coli*,^98^ quite a change from the mitochondria and the MAS that I studied for my PhD. Ochoa was an impressive tall man and he looked like a Spanish don who had just stepped out of an El Greco painting. Although an imposing figure, he was a gentle, helpful supervisor in the lab and working long hours, notwithstanding his Nobel Prize. He is usually known for his work on nucleic acids and the genetic code, but he had a long and productive career in metabolism before switching to nucleic acids.

m It is remarkable that most papers in this field overlook the need for the glutamine‐fructose transamidase and seem to assume that there is glutaminase activity in the mammalian cytosol, even in reviews that prominently feature attempts to inhibit tumor growth using inhibitors of glutaminase,^100^, ^101^ or in articles that focus on glutamine metabolism.^102^

n Although Crabtree^116^ confirmed that tumors had a relatively high aerobic glycolysis, he was already very skeptical of Warburg's postulate that “a disturbance in respiration as being the fundamental cause of the development of aerobic glycolysis. In many of the tumors, the respiration is very high, exceeding that of any tissue, …”^116^ And that in 1929! Obviously, the influence of powerful Warburg was so large before World War 2 that such skepticism remained invisible.

o My thesis project was not a very imaginative one, since it was known in 1958 already that some tumors do not have any aerobic glycolysis at all and that tumor mitochondria look pretty normal, when properly isolated. Indeed, I found Ehlich ascites cell mitochondria to be similar to rat‐liver and ‐heart mitochondria in every aspect that I could investigate with the methodology available around 1960 (see Reference 1). The uncoupling compound found in aged mitochondria turned out to be mainly unsaturated long‐chain fatty acids,^119^ an artifact generated in unhealthy isolated mitochondria. This should not be taken as criticism of my thesis supervisor Bill Slater, who was in many respects the ideal supervisor, conscientious, very knowledgeable, highly supportive, and scrupulous. The fact that about half the papers I published as his student only have my name on it is typical. He was critically involved, but found his own contribution to my work insufficient to merit co‐authorship. The cancer project was a side‐line and the (rare) instance where Slater was taken in by a paper in the respectable JBC that looked OK, but turned out to be a total misinterpretation of sloppy science.
